# MRI-based or CT-based patient-specific instrumentation in Total knee Arthroplasty: How do the two systems compare?

**DOI:** 10.1186/s42836-019-0020-6

**Published:** 2020-01-14

**Authors:** Dong-Geun Kang, Kang-Il Kim, Jung-Kwon Bae

**Affiliations:** Department of Orthopaedic Surgery, Kyung Hee University Hospital at Gangdong, 892 Dongnam-ro, Gangdong-gu, Seoul, 134-727 South Korea

**Keywords:** Total knee arthroplasty, Patient specific instrumentation, Magnetic resonance image, Computed tomography

## Abstract

**Background:**

Patient-specific instrumentation (PSI) has been introduced into total knee arthroplasty (TKA) to improve accuracy in restoration of alignment. PSI in TKA refers to custom-made cutting jigs manufactured according to anatomic configuration of the patient’s bone based on preoperative magnetic resonance imaging (MRI) or computed tomography (CT) scans. The purpose of this study was to compare the MRI- or CT-based PSI to see if they could reproduce accurate bone resection and postoperative outcomes.

**Methods:**

Seventy-one patients who received elective TKA using a PSI system for primary osteoarthritis with varus deformity were prospectively enrolled for this study. We randomly allocated those patients to MRI-based PSI group (36 patients) and CT-based PSI group (35 patients). The actual resection thickness and planned resection thickness by preoperative PSI electronic program were compared between the two groups. Radiographic findings of the postoperative limb alignment, three-dimensional position of the implants, and related complications were also evaluated. Clinical evaluation was also performed before and 2 years after the surgery.

**Results:**

There were no significant differences in the resection thickness in femur and tibia between actual resection and planned resection in both groups. Furthermore, there were no significant differences between two groups in terms of coronal, sagittal and rotational alignment of the components. All clinical assessments revealed no differences between two groups 2 years after the operation. No specific complication related to PSI was observed.

**Conclusions:**

Although MRI allows for visualization of cartilage, MRI-based PSI system did not show better accuracy in predicting the thickness of bone resection than CT-based PSI. Moreover, there were no differences in radiographic and clinical outcomes between the two groups.

## Background

Patient-specific instrumentation (PSI) has been introduced into total knee arthroplasty (TKA) as a new technology for improving accuracy in restoration of alignment and biomechanics of the lower limb [1–4]. PSI in TKA refers to custom-made cutting jigs manufactured according to the patient’s anatomic configuration of distal femur and proximal tibia based on preoperative magnetic resonance imaging (MRI) or computed tomography (CT) scans [5–8]. MRI- and CT-based PSI systems are available from various manufacturers for preoperative planning. MRI gives concrete form to articular cartilage without the risk of radiation exposure, but it is relatively expensive and requires longer scan time than CT. In contrast, CT enables accurate identification of the contour of the femur and tibia for shorter scan time [9], but it cannot provide information on the articular cartilage and carries the risk of radiation exposure. As a result, there might be some discrepancies between the thickness of bone resection proposed by MRI- or CT-based PSI system and the actual thickness of bone cutting. MRI has the theoretical advantage of full visualization of articular cartilage and is supposed to be more accurate in predicting bone resection thickness in TKA than the CT-based PSI system. Previous studies compared PSI with the conventional instrumentation and computer-assisted navigation system in terms of its efficacy in improving the accuracy of TKA [10–12]. Although PSI has been the focus of many recent researches [6, 12–16], there have been a few clinical studies comparing MRI- and CT-based PSI systems in preoperative planning [17–19]. However, to our knowledge, this would be the first study to examine the accuracy of bone resection by preoperative planning for two imaging modalities in PSI system. The purpose of this study was to know whether the MRI- or CT-based PSI could reproduce accurate bone resection and better postoperative outcomes. Therefore, we tried to know whether the MRI-based PSI that reflects the cartilage layer would provide more precision in TKA than the CT-based PSI. We hypothesized the MRI-based PSI would be more accurate for resection thickness of lateral aspect of proximal tibia where the cartilage is relatively sound in varus knee and the two systems would have no difference in the resection of the medial femoral condyle where the cartilage would be generally worn in varus knee. In addition, we compared the radiographic findings and clinical outcomes between the groups.

## Methods

### Study design

**Fig. 1:**
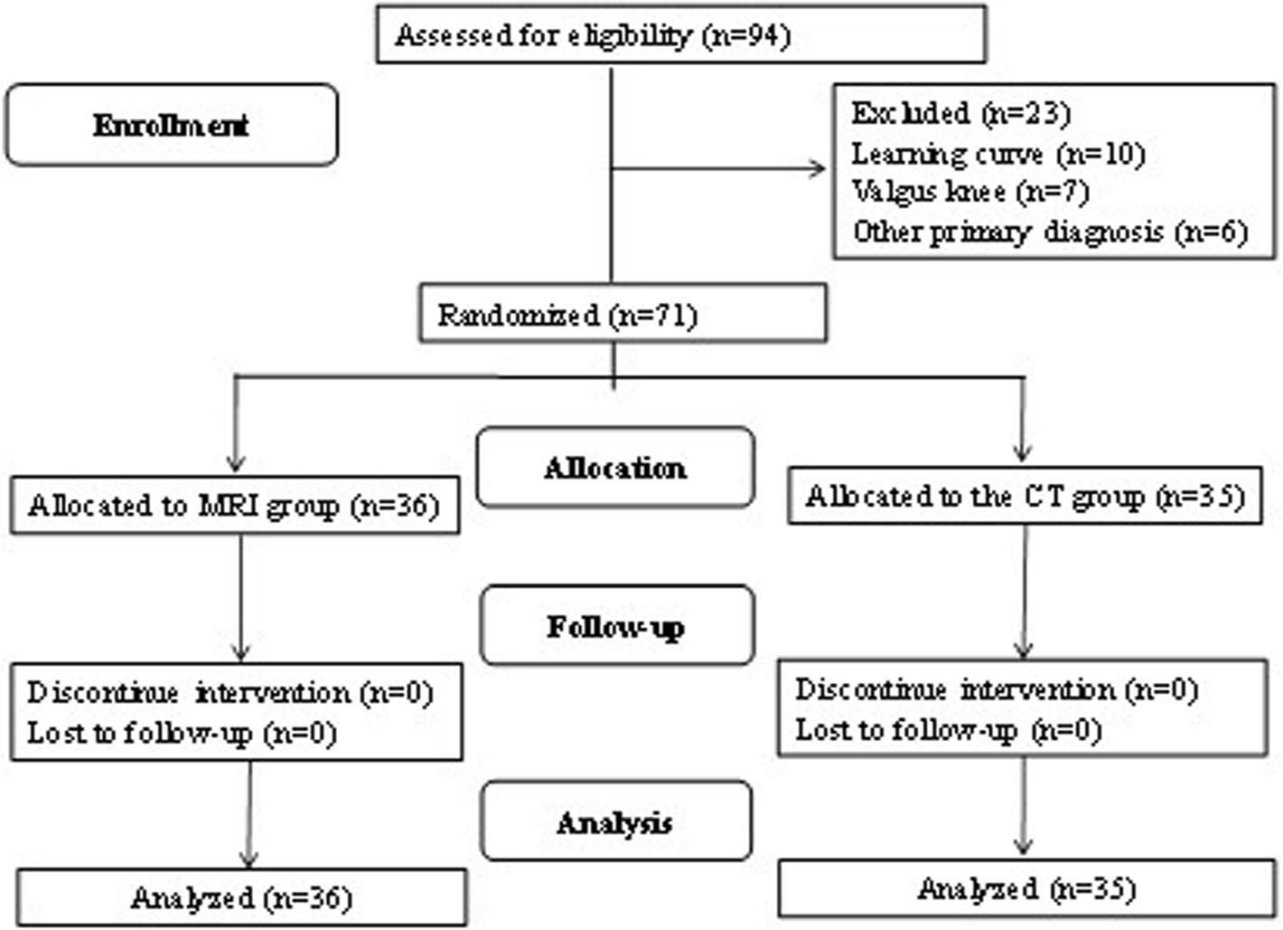
CONSORT flow diagram of randomization and follow-up of patients

This was a prospective study of PSI-guided TKAs performed by a single surgeon (KIK). The institutional review board granted approval for this study. Written informed consent was obtained from all patients before the surgery. Of the patients who had been scheduled for TKA for the treatment of primary osteoarthritis only with varus deformity, those who had been waiting 6 weeks for TKA using an MRI-based or CT-based PSI system and had consented to the relatively new technique were enrolled. Patients with primary osteoarthritis with valgus deformity, rheumatoid arthritis, hemophilic arthritis, posttraumatic arthritis, other inflammatory arthritis, or a history of previous high tibial osteotomy were excluded from the study. Therefore, 71 TKA candidates were assigned, using a computer-derived randomization table with block sizes of four, to receive an MRI scan (MRI group; *N* = 36) or a CT scan (CT group; *N* = 35) (Fig. 1). In all patients, the Signature™ Personalized Patient Care System (Biomet Inc., Warsaw, Indiana) manufactured using Materialise® software (Leuven, Belgium) was used. There were no significant differences between the groups with regards to age, gender, body mass index, degree of preoperative deformity (Table 1). The follow-up assessment was conducted 2 years after the surgery in all patients without exception.

**Table 1:** Demographic data

Parameter	MRI group (*N* = 36)	CT group (*N* = 35)	*P* Value
Age (year)^a^	69.1 ± 5.9	68.5 ± 7.0	0.685
Sex (male: female)	5:31	1:34	0.199
BMI (kg/m^2^)^a^	27.1 ± 4.0	27.1 ± 3.3	0.990
Preoperative MA (°)^a^	−9.9 ± 4.8	−10.4 ± 4.8	0.673
Preoperative FTA (°)^a^	−3.9 ± 4.5	−3.9 ± 5.2	0.963

^a^The values are given as the mean and the standard deviation

*BMI* Body mass index, *MA* Mechanical axis, *FTA* Femorotibial angle

### Preoperative planning and PSI manufacturing

In the MRI group, preoperative MRI data acquisition was performed using an MRI scanner (Achieva 3.0 T, Philips, Netherlands) according to the PSI system manufacturing protocol: 1-mm high-resolution slices of the knee and selective 5-mm spot images of the hip and ankle were acquired to measure the MA and to correct rotation of the lower extremity. The MRI images in DICOM format (Digital Imaging and Communications in Medicine) were uploaded onto the software to construct three-dimensional (3D) models. Then, the patient-specific cutting jigs were generated based on the surgeon’s predetermined default settings. The femoral component rotation was set to be parallel to the trans-epicondylar axis, perpendicular to the MA on the coronal plane, and in 3° flexion on the sagittal plane, and medial distal resection was set at 9 mm. The default settings for tibial component alignment were perpendicular to the MA on the coronal plane (0° varus/valgus) and at a 3° posterior slope on the sagittal plane, and tibial resection was set at 10 mm below the highest point of the lateral tibia. The tibial component rotation could not be adjusted by the software.

In the CT group, preoperative CT data acquisition was performed using a CT scanner (Brilliance 64 Channel Multi Detector Computed Tomography, Philips, Israel) according to the same PSI system manufacturing protocol: 1.25-mm slices of the lower extremity were obtained to measure the mechanical axis (MA) and to correct rotation of the lower extremity. The CT images in DICOM format were processed by the same software to reconstruct 3D models. Since most varus knees in primary osteoarthritis have a relatively sound lateral compartment and a worn medial compartment, the default setting values were identical to those used for the MRI-based PSI in the medial bone cut, lateral cutting thickness of proximal tibia was set at 8 mm below the highest point of the lateral tibia. We presumed it would be 10 mm of tibial cutting by compensating for the cartilage which could not be seen on CT scans [20, 21]. The predetermined values for the implant size, knee alignment, and thickness of bone resection in both groups were reviewed for potential adjustment. Upon approval of the preoperative plan, the PSI manufacturer was requested to produce cutting jigs and bone models designed to represent the patient’s anatomical configuration of the femur and tibia. The bone models and cutting jigs were made of polyamide and fabricated using rapid prototyping [4].

### Operative technique

All the operations were performed under general anesthesia and tourniquet control by a senior surgeon using a minimally invasive mid-vastus approach. The patient-specific cutting jigs were used for bone resection in all knees: without osteophyte removal, the cutting jigs were placed on the femur and the tibia, ensuring stable fitting without toggling. By using a modified gap technique, the distal femur was resected, which was followed by proximal tibial bone cutting and femoral chamfer resection by employing PSI cutting jigs. Except for the use of PSI instead of the conventional instrument for bone resection, overall surgical procedure was identical to the routine conventional method. All bone cuts were made using conventional 1.27-mm saw blades. All patients received the posterior-stabilized cemented total knee system (Vanguard™, Biomet, Warsaw, Indiana). A suction drain insertion, pneumatic pump application to prevent deep vein thrombosis, multimodal pain managements, and postoperative rehabilitation protocol were identical in all patients.

### Assessment

After bone resection using a patient-specific cutting jig, the actual thickness of resected bone was measured in 0.1 mm increments by the Vernier caliper (B. Braun-Aesculap, Tuttlingen, Germany) and compared with the planned resection thickness preoperatively using the PSI program. In the MRI group, the intraoperative cutting thickness was compared directly with the preoperatively planned thickness, whereas the presumed thickness of cartilage (2 mm) was added to actual thickness of resected bone from the lateral condyles in the CT group [20, 21]. Finally, the thickness of saw blade (1.27-mm) was added to every resected bone in all cases.

On the radiographic assessment, the MA of the lower extremity and the coronal and sagittal alignments of the femoral and tibial components were measured on the anterior and lateral weight-bearing radiographs of the knee and the standing long-leg radiograph before the surgery and 2 years after the surgery. The measurements were performed using a picture-acquiring communication system (PACS, Infinitt Healthcare, Seoul, Korea), and the data were recorded to one decimal place. The MA was defined as the angle formed by the femoral MA (a line between the center of the femoral head and the center of the knee) and the tibial MA (a line between the center of the ankle and the center of the knee): 0° was regarded as neutral, a varus alignment was assigned a negative value, and a valgus alignment was assigned a positive value. The coronal femoral angle (α) and the coronal tibial angle (β) were assessed on the anteroposterior weight-bearing radiograph, whereas the sagittal femoral angle (γ) and the sagittal tibial angle (δ) were measured on the lateral radiograph [22]. The femoral component rotation was assessed on the postoperative 3D CT scan. The femoral component rotation was defined as the angle difference between the posterior condylar axis of the femoral component and the surgical epicondylar axis [23]. An outlier was defined as ±3° deviation from the ideal alignment. The tibial component rotation was not assessed postoperatively. We believed PSI would have no direct influence on the postoperative rotational alignment of the tibial component because it was not included in the preoperative planning and the optimal alignment was determined intraoperatively based on the assessment of knee flexion/extension after trial insertion. Measurements were performed by two blinded, independent observers, twice each, with a minimum interval of 2 weeks.

For clinical assessment, clinical scores before the surgery and 2 years after the surgery were compared between the two groups. Clinical assessment included the Knee Society knee score [24] and the 36-item Short Form Survey [25] in all patients.

### Statistical analysis

Continuous variables including radiographic measurements, and differences in resected bone thickness were compared between groups with Student’s *t*-test or Mann-Whitney U test (for data that were not normally distributed). Pearson’s chi-square test, Fisher’s exact test were used to compare categorical variables between the two groups, such as the incidence of outliers of MA and femoral component rotation. All statistical analyses were performed with SPSS for Windows, version 12.0 (SPSS, Chicago, Illinois). *P* values < 0.05 were considered statistically significant. Inter- and intraobserver reliability was assessed using the intraclass correlation coefficient (ICC). The ICCs were classified as follows: poor, less than 0.4; marginal, 0.4–0.75; and good, above 0.75.

## Results

The absolute differences between the planned resection thickness and the actual resection thickness in the distal femur and the proximal tibia were not significantly different between the MRI group and the CT group (Table 2). The mean postoperative MA of the lower limb and the prevalence of outliers with reference to the MA did not differ between the two groups (*p* > 0.05) (Table 3). Moreover, there was no notable intergroup difference in the mean sagittal and coronal alignments of the femoral and tibial components 2 years after TKA. The mean rotational alignment of the femoral component was 1.2° external rotation in the MRI group and 1.1° external rotation in the CT group (*p* > 0.05). The incidences of outliers were also similar between the groups. The intraobserver ICC for each variable was 0.863–0.935 and the interobserver ICC was 0.807–0.836, indicating high agreement between the observers and measurements. There was no difference in mean preoperative and postoperative Knee Society knee and functional scores, 36-item Short Form Survey between the two groups 2 years after surgery (Table 4). No patients required reoperation or re-hospitalization following TKA, and there were no perioperative complications, including deep or superficial infection, symptomatic deep vein thrombosis or pulmonary embolisms, and periprosthetic fracture until the latest follow-up.

**Table 2:** Comparison of absolute differences between the planned and actual bone resections in both groups

(mm)	MRI group (*N* = 36)			CT group (*N* = 35)			MRI vs CT
	Planned	Actual	Difference^†^	Planned	Actual	Difference^†^	*P* Value
DFM	9.0 ± 0.7	8.5 ± 1.2	0.9 ± 0.7	8.7 ± 0.9	9.0 ± 1.6	1.2 ± 0.7	0.192
DFL	9.1 ± 1.1	8.3 ± 1.6	1.4 ± 0.9	9.3 ± 1.6	8.4 ± 1.9	1.6 ± 1.0	0.292
PFM	9.3 ± 0.6	9.9 ± 1.3	1.1 ± 0.9	8.8 ± 0.5	8.9 ± 1.8	1.4 ± 0.9	0.173
PFL	7.8 ± 1.4	7.2 ± 1.5	1.3 ± 0.9	8.6 ± 1.2	7.3 ± 1.5	1.6 ± 0.8	0.062
TM	2.5 ± 1.8	2.9 ± 1.0	1.1 ± 0.9	2.1 ± 1.3	2.7 ± 0.8	1.1 ± 0.9	0.902
TL	10.0 ± 0.4	9.4 ± 1.3	1.3 ± 0.8	10.3 ± 0.7	9.5 ± 1.4	1.2 ± 0.8	0.730

*The values are given as the mean and the standard deviation. ^†^paired *t*-test

*DFM* Distal femoral medial side, *DFL* Distal femoral lateral side

*PFM* Posterior femoral medial side, *PFL* Posterior femoral lateral side

*TM* Tibial medial side, *TL* Tibial lateral side

**Table 3:** Postoperative radiographic measurements

Parameter	MRI group (*N* = 36)	CT group (*N* = 35)	*P* Value
Mechanical axis (MA)^a^	−2.1° ± 2.1°	−2.1° ± 2.5°	0.996
MA outlier (> 3°)^b^	7 (19.4%)	8 (22.9%)	0.778
Coronal femoral angle (α)^a^	94.9° ± 1.1°	94.7° ± 2.0°	0.747
Coronal tibial angle (β)^a^	90.0° ± 2.0°	90.1° ± 1.5°	0.909
Sagittal femoral angle (γ)^a^	5.0° ± 2.9°	4.3° ± 2.8°	0.282
Sagittal tibial angle (δ)^a^	86.2° ± 2.8°	85.6° ± 3.0°	0.420
Femoral external rotation (ER)^a^ to transepicondylar axis	1.2° ± 1.1°	1.1° ± 1.4°	0.810
Femoral ER outlier (> 3°)^b^	1 (2.8%)	3 (8.6%)	0.357

^a^The values are given as the mean and the standard deviation

^b^Outlier values are given as the number of knees, with the percentage in parentheses

**Table 4:** Clinical outcomes between MRI- versus CT-based PSI after total knee arthroplasty

Parameter	MRI group (*N* = 36)	CT group (*N* = 35)	*P* Value
Knee Society knee score			
preoperative	44.3 ± 18.6	42.8 ± 16.1	0.725
2-year follow-up	92.7 ± 10.7	92.2 ± 11.8	0.815
Knee Society function score			
preoperative	48.7 ± 10.8	46.9 ± 10.4	0.614
2-year follow-up	91.9 ± 15.8	89.7 ± 12.9	0.586
SF-36 (physical function)			
preoperative	30.9 ± 9.7	37.2 ± 8.8	0.436
2-year follow-up	47.5 ± 8.5	47.2 ± 8.5	0.539
SF-36 (mental health)			
preoperative	48.2 ± 4.6	50.7 ± 5.3	0.487
2-year follow-up	56.5 ± 10.1	60.8 ± 11.9	0.639

## Discussion

This study demonstrated that there were no significant differences between the MRI- and CT- based PSI systems in following investigated parameters: 1) the differences between the planned resection thickness and the actual resection thickness in the distal femur and the proximal tibia; 2) the mean postoperative MA of the lower limb and the prevalence of outliers with reference to the MA; 3) the mean postoperative alignments and short-term clinical outcomes.

The differences between the planned resection thickness and the actual resection thickness in the distal femur and the proximal tibia were not significantly different in both studied groups in this study. In the current study, the primary osteoarthritic knee with varus deformity alone was an inclusion criterion. Therefore, we assumed that the medial femoral cartilage was mostly worn out, whereas the cartilage thickness in the lateral tibial condyle was relatively sound in all study participants. In the CT group, we added 2 mm of presumed cartilage thickness to the bone thickness of lateral tibial resection to compensate for the cartilage layer surrounding the subchondral bone that was not visualized on CT. In the MRI group, there was no need to make such adjustments because MRI had the ability to represent the cartilage layer. The study showed no significant difference between the two groups in terms of the thickness of bone resection of the lateral aspect of the femur. Therefore, we recommend adding approximately 2 mm of cartilage thickness to the value proposed by the CT-based PSI system in determining the resection thickness of the lateral aspects of the tibia in patients with varus deformity [20, 21]. In both MRI and CT groups, about 1 mm of the mean absolute difference was noted between the planned resection thickness and the actual thickness of the resected bones on the worn medial side and the relatively intact lateral side, indicating that the accuracy of the PSI system was high. Furthermore, no statistically significant intergroup difference was observed, and the difference was within the margin of error. Hafez et al. [26] reported that the mean bone resection error was 0.32 mm (maximum, 1 mm) in 16 cadavers and 29 plastic knee specimens after TKA using a CT-based PSI. This experimental result also supports our current study with in vivo settings. However, that study did not consider the thickness of invisible cartilage.

Besides the assessment of resection thickness of femur and tibia using PSI system, there have been several studies comparing CT- and MRI-based PSI system in TKA [17–19]. Two studies [17, 19] compared the accuracy of MRI- and CT-based PSI but they used PSIs from two different manufacturers and different types of the guide. One randomized clinical trial by Pfitzner et al. [17] compared the accuracy of MRI- and CT-based PSI with conventional instrumentation and with each other in TKAs. They found the PSI system increased accuracy compared with conventional instrumentation and that MRI-based PSI was more accurate compared with CT-based PSI, regarding coronal mechanical limb axis, but differences were only subtle and of questionable clinical relevance. And Asada et al. [19] reported that both systems would result in the same accuracy on three planes but high inaccuracy on the sagittal plane on radiographs. While, Silva et al. [18] evaluated the same PSI systems used in our study and reported improved tibial component rotation with MRI-based PSI as compared to CT-based PSI. However, they only assessed postoperative rotation of femur and tibial components. Furthermore, Frye et al. [27] reported better coronal alignment with MRI-based PSI than CT-based PSI. In the current study, the mean coronal and sagittal alignments of the femoral and tibial components were not significantly different between two groups. The rotational alignment of the femoral component has been investigated in several studies. Heyse and Tibesku [28] reported that the incidence of outliers in rotational alignment was remarkably low (2.2%) in a postoperative MRI analysis of 46 TKAs using an MRI-based PSI, whereas Noble et al. [12] observed outliers in 23.2% of the 60 knees after TKA using an MRI-based PSI. However, it should be noted that these studies were comparison between the PSI system and the conventional equipment. In the current study, no significant difference was found in the rotational alignment and the incidence of outliers between the MRI and CT groups.

MRI and CT may have their own strengths and limitations when used for creation of PSI system, and a discrepancy appears to unavoidably develop between the bone model and the actual shape and size of the patient’s femur and tibia due to various stages of measurement and manufacturing processes [8, 9]. In an initial animal study on PSI system, bone models generated from CT scans have been shown to be more accurate and their external surface boundaries smoother and freer from distorting artifacts than MRI-based models in 10 ovine knees [8]. In contrast, MRI scans enabled more accurate component alignment in knees in which the articular cartilage was worn away than CT scans [9]. And, based on the cartilage surface estimated by the surgeon with CT-based PSI, deviation from the real joint surface is possible, which can result in a poorer fit. However, there was no difference on the three planes between the MRI and CT groups in our study. It is likely that the fitting of the CT-based PSI guide on the articular surface would be sufficient, despite the loss of the contract area.

This study also had several limitations. First, the cost-effectiveness of PSI was not assessed. However, the current study mainly focused on the comparison of accuracy between the preoperatively planned cutting thickness and the intraoperative actual cutting thickness in both CT- and MRI-based PSI systems rather than the effectiveness of the system itself. Moreover, as individual countries have different policies concerning the price of using it and different degrees of convenience in terms of using MRI/CT, comparison in such manner would apparently be considered subjective. Second, because the CT-based and MRI-based PSI were the products of the same manufacturer; therefore, it might be difficult to generalize our results to other PSI systems. Third, the number of subjects was relatively small. This study was planned prospectively but, the sample size was not researched to calculated sample size. And finally, we did not evaluate the PSI against conventional instrument in TKA.

Despite the advantage of full visualization of the cartilage layer, the MRI-based PSI did not show better accuracy than the CT-based PSI in terms of planned resection thickness in TKA. Therefore, we recommend that a preoperative decision on whether MRI or CT will be used for creation of PSI should be based on the availability of an imaging modality in the hospital/country system, the risk of radiation exposure and preference of a surgeon or a patient. In addition, we suggest that, in the planning of TKA by using a CT-based PSI system in a varus knee, adjustment should be made in preoperative planning to add approximately 2 mm of invisible cartilage thickness in determining the extent of resection of the lateral tibial condyle where the cartilage will be relatively well maintained.

## Conclusion

Although MRI provides visualization of cartilage, MRI-based PSI system did not show better accuracy in predicting the thickness of bone resection than CT-based PSI. Moreover, there was no difference in radiographic findings and clinical outcomes between the two groups. Future studies should compare the two modalities in terms of costs and burden of resources.

## Data Availability

The datasets used and/or analysed during the current study are available from the corresponding author on reasonable request.

## Abbreviations

3D: Three-dimensional
CT: Computed tomography
ICC: Intraclass correlation coefficient
MA: Mechanical axis
MRI: Magnetic resonance imaging
PSI: Patient-specific instrumentation
TKA: Total knee arthroplasty
